# Electric Double Layers with Surface Charge Regulation Using Density Functional Theory

**DOI:** 10.3390/e22020132

**Published:** 2020-01-22

**Authors:** Dirk Gillespie, Dimiter N. Petsev, Frank van Swol

**Affiliations:** 1 Department of Physiology and Biophysics, Rush University Medical Center, Chicago, IL 60612, USA; dirk_gillespie@rush.edu; 2 Department of Chemical and Biological Engineering and Center for Micro-Engineered Materials, University of New Mexico, Albuquerque, NM 87131, USA; dimiter@unm.edu

**Keywords:** electrolyte solution, double layer, charge regulation, ion correlations

## Abstract

Surprisingly, the local structure of electrolyte solutions in electric double layers is primarily determined by the solvent. This is initially unexpected as the solvent is usually a neutral species and not a subject to dominant Coulombic interactions. Part of the solvent dominance in determining the local structure is simply due to the much larger number of solvent molecules in a typical electrolyte solution.The dominant local packing of solvent then creates a space left for the charged species. Our classical density functional theory work demonstrates that the solvent structural effect strongly couples to the surface chemistry, which governs the charge and potential. In this article we address some outstanding questions relating double layer modeling. Firstly, we address the role of ion-ion correlations that go beyond mean field correlations. Secondly we consider the effects of a density dependent dielectric constant which is crucial in the description of a electrolyte-vapor interface.

## 1. Introduction

The occurrence of electrolyte solutions meeting solid (or liquid), materials is ubiquitous. The behavior of the resulting interfaces is key in the fields of surface and colloid science, electrochemistry and corrosion and soft materials and biomaterials. These interfaces are subject to both physical and chemical interactions with the fluid. Examples include chemical reactions between surface terminal groups and certain solution components, physical adsorption or desorption of charges at the interface [1,2]. All these interfacial phenomena may manifest themselves in the accumulation of surface charge at the interface. Consequently, the electrostatic surface potential will change. As a result, mobile charges in the vicinity will redistribute to lower the total free energy. Thus, surface chemistry effects propagate into the neighboring phase. The entire system, consisting of charged interface and locally distributed mobile charges and electrostatic potential is referred to as the electric double layer (EDL). The double layer name has historical roots, reflecting the initial notion that the charged interface with the electrolyte solution can be represented by a simple capacitor, where the surface charges and the solution counter-charges are placed on two well-defined planes in space [3]. This is certainly not an accurate representation of the physical situation, as entropic effects cause the charges to distribute themselves in space, away from a strict capacitor plane. An alternative description was suggested by both Gouy and Chapman [4,5,6] who realized that the EDL had diffuse aspects. They suggested a model based on Maxwell theory of electrodynamics, relating the potential Ψ(r) to the charge density distribution $\rho_{e}\left(r\right)$.

In parts of this paper we account for the presence of the solvent but we stress that this accounting is incomplete. It captures the important basic aspects of (1) excluded volume and (2) solvent-ion attractions (i.e., ion solvation) through the the Lennard-Jones (LJ) interactions. For instance, the effects of solvent orientation and solvent polarizability are not included in any detail, but rather mimicked through an *effective* LJ interaction. Similarly, the network structure of a solvent like water, say, including the quantum nature of hydrogen bonding are not captured at this time with current classical density functional theory (cDFT) techniques. Some authors have criticized models that do not include all known aspects of the solvent. We point out that there is always great value in the usage of simple models as their strength is to highlight and explore fundamental aspects and to provide insight into the phenomena of interest (e.g., the behavior of EDLs). In addition, there are recent approaches (see References [7,8,9]) that use experimentally measured ion solvation energies to improve the values used to approximate the LJ interactions, say. Such a strategy can address ion-specific effects that experiments have established.

The inclusion of more detail is then made when it becomes clear that the simple model approach fails. A nice illustration of such a situation can be found in the work of Wilson and Madden [10], who showed that to obtain the correct crystal structure for simple divalent salts like ${\mathrm{MX}}_{2}$ it is necessary to include ion polarizability into the lowest free energy calculation.

Having recognized the strengths and weaknesses of density functional theory (DFT) it is worthwhile to stress that molecular simulation is the more convenient approach to explore the effects of solvation details including polarizability, orientation, hydrogen bonding and quantum effects.

The remainder of this paper is arranged as follows. In the next section we introduce the chemical boundary condition known as charge regulation and then in the next section we introduce the details of density functional theory that includes a neutral solvent component. We then discuss the role of screening ion-ion correlations that go beyond mean field. Finally, we address the role of the dielectric permittivity at an interface and introduce the Oleksy-Hansen method of a permittivity that depends on the local (coarse-grained) density. In the results section we illustrate the effects of charge regulation and the solvent inclusion by looking at the net charge distribution in a double layer and the corresponding electric potential distribution for three different wetting cases. We then illustrate the effects of including screening ion correlations for the primitive model and show how it can produce charge inversion. The latter is an impossibility in the Poisson-Boltzmann approximation. Finally, we illustrate the effects of ion correlations on fluid flow. The paper then concludes with a conclusions section.

## 2. Charge Regulation

Poisson’s equation [11] is an example of a second-order partial differential equation (pde),

(1) $$\begin{array}{cc} \; & \nabla^{2}\Psi =-\frac{\rho_{e}}{\varepsilon_{r}\varepsilon_{0}}. \end{array}$$

When the right-hand side is equal to zero the equation is reduced to Laplace’s equation. Poisson’s equation describes the variation of the electrostatic potential, Ψ, in space, given a spatial distribution of charges, $\rho_{e}\left(r\right)$. In the study of electrolyte solutions the situation is slightly different, there we seek to find the equilibrium distribution of charges.

This is a far more complex problem. Two familiar examples are the ionic distribution in a bulk electrolyte fluid and the distribution of ions in an electrical double layer. The first allows the determination of ionic activity coefficients whereas the second example leads to the prediction of forces acting between charged surfaces, as encountered in colloid stability studies.

To make progress with solving the Poisson equation one requires a simplification. This can be accomplished by postulating a relationship between the charge distribution and the electrostatic potential. Inspired by an ideal gas in an external field the approximation used stipulates that the density distribution is proportional to the Boltzmann factor of the potential. This approximation becomes exact in the low-density limit. Substituting this expression into the Poisson equation produces the so-called Poisson-Boltzmann (PB) equation, a nonlinear second order partial differential equation for the potential:

(2) $$\begin{array}{c} \rho_{e}=\sum_{i}\rho_{i}^{0}q_{i}\exp\left(\frac{-q_{i}\Psi }{k_{B}T}\right), \end{array}$$

where $\rho_{i}^{0}$ is the number density of ionic species *i* and $q_{i}$ is the ionic charge including the sign (in units of *e*). The charge screening is characterized by the inverse Debye length κ is defined as,

(3) $$\begin{array}{cc} \; & \kappa^{2}=\frac{1}{\varepsilon_{r}\varepsilon_{0}k_{B}T}\sum_{i}q_{i}^{2}\rho_{i}^{0}. \end{array}$$

Debye and Huckel solved the *linearized* PB equation for an electrolyte solution whereas Gouy and Chapman solved the linearized PE equation for a charged surface. To solve the latter PB equation requires a boundary condition (bc). Natural and numerically convenient choices for bc are the potential at the surface (i.e., Dirichlet condition) or the spatial derivative of the potential at the surface (i.e., the surface charge, known as the Neumann condition).

In 1971, Parsegian and Ninham [12] proposed a radically different approach that is more true to the actual local chemistry. They drew attention to the fact that charged surfaces acquire their surface as a result of an ionizable surface group. That ionization is, ultimately, a chemical reaction where a neutral surface group splits of an ion leaving a charged surface group. A simple example would be a deprotonation of a surface group AH, viz.

(4) $$\begin{array}{cc} {\mathrm{AH}}_{2}^{+}+\mathrm{BH} & ⇌\mathrm{AH}+{\mathrm{BH}}_{2}^{+},{\mathrm{pK}}_{+}=-\log_{10}K_{+}\\ \mathrm{AH}+\mathrm{BH} & ⇌A^{-}+{\mathrm{BH}}_{2}^{+},{\mathrm{pK}}_{-}=-\log_{10}K_{-}. \end{array}$$

This leaves a negatively charged surface. An equilibrium constant K (or equivalently a Gibbs free energy change of reaction, ΔG) relates the local concentrations of the reactants, here $AH,A^{-}$ and $H^{+}$, where it is understood that both AH and $A^{-}$ are surface bound. In what follows, ΔG will typically be taken from the literature or set equal to the value in the bulk.

The relevant local concentration of the proton is that at the surface. If the surface is represented by a hard wall then this can be the contact density at the wall. On the other hand, if the wall is manifested as a smooth potential there is no contact density and a choice needs to be made. In previous work we have selected a density average over a narrow range, we shall employ that definition here. Alternatively, one could consider a profile based equivalent to the bulk *y*-function (i.e., y(r)=g(r)exp(ϕ(r)/kT)) [13], that is

(5) $$y\left(z\right)=\rho \left(z\right)e^{V_{ext}\left(z\right)/kT}.$$

This is simply a smooth and continuous extension of the profile into the wall region. The contact density must then be measured at an effective hard wall position. The reader is referred to Reference [2] for more details regarding the use of the *y*-function in reactive systems and the use of y(z) in interfacial systems.

The Parsegian and Ninham approach replaces a physical bc (constant surface charge or constant surface potential) with a chemical bc, expressed in terms of a surface reaction’s equilibrium constant (Ka or pKa). It is known as charge regulation (CR) as the surface charge is regulated by Ka (or pKa). This implies that both the surface charge and the surface potential are no longer input parameters but instead found from the solution of the electrostatic double layer problem. CR was originally proposed for a PB approach to double layers. More recently, Fleharty et al. [14] took the step to implement charge regulation in a DFT formulation of a double layer.

## 3. The Grand Thermodynamic Potential

EDLs involve multicomponent solutions, containing charged (ionic) and, if included, neutral (solvent) species. These species are subjected to the effects of the field exerted by the interface with the substrate. The field has electrostatic and van der Waals components, which lead to a variety of interactions with the fluid. In addition, the solution components (M) in the fluid interact with each other. All of these interactions are easily incorporated in a cDFT model.

The electrolyte solution is described in terms of a grand thermodynamic potential functional, which for single flat EDL reads [14,15,16,17,18,19,20]

(6) $$\begin{array}{ll} \Omega \left[\left\{\rho_{i}\left(z\right)\right\}\right]=F^{id}\left[\left\{\rho_{i}\left(z\right)\right\}\right]+F_{HS}^{ex}\left[\left\{\rho_{i}\left(z\right)\right\}\right] & +F_{long}^{ex}\left[\left\{\rho_{i}\left(z\right)\right\}\right]+F_{SC}\left[\left\{\rho_{i}\left(z\right)\right\}\right]+\\ & 2\pi \sum_{i=1}^{M}\int RdR\int dz\;\rho_{i}\left(R,z\right)\left[V_{i}^{ext}\left(R,z\right)-\mu_{i}\right]. \end{array}$$

The first term on the right hand side of Equation (6) corresponds to the ideal contribution to the free energy,

(7) $$F^{id}\left[\left\{\rho_{i}\left(z\right)\right\}\right]=2\pi \;k_{B}T\sum_{i=1}^{M}\int RdR\int dz\;\rho_{i}\left(R,z\right)\left\{\ln\left[\lambda_{i}^{3}\rho_{i}\left(R,z\right)\right]-1\right\},$$

where $\lambda =\sqrt{h^{2}/\left(2\pi m_{i}k_{B}T\right)}$ is the thermal de Broglie wavelength, *h* is Planck’s constant, $m_{i}$ is the mass of species “*i*” and $\rho_{i}\left(z\right)$ is the local density of component “*i*” along the *z* coordinate normal to the wall. The radial coordinate R runs parallel to the EDL interface with the substrate.

The excess free energy consists of hard-sphere and long-range parts. The hard-sphere contribution is based on the derivation of Rosenfeld [21,22] and reads

(8) $$F_{HS}^{ex}\left[\left\{\rho_{i}\left(z\right)\right\}\right]=2\pi \;k_{B}T\int RdR\int dz\;\Phi_{HS}\left\{n_{\alpha }\left(R,z\right)\right\}.$$

$\Phi_{HS}\left\{n_{\alpha }\left(R,z\right)\right\}$ is the hard-sphere reduced free energy and $n_{\alpha }\left(R,z^{\prime }\right)=n_{\alpha }\left(r\right)$ (r being the position vector) is the weighted local density.

(9) $$\Phi_{HS}\left\{n_{\alpha }\left(r\right)\right\}=-n_{0}\log\left(1-n_{3}\right)+\frac{n_{1}n_{2}-n_{1}\cdot n_{2}}{1-n_{3}}+\frac{n_{2}^{3}-3n_{2}n_{2}\cdot n_{2}}{24{\left(1-n_{3}\right)}^{2}}.$$

Remarkably, the functional form of the reduced free energy $\Phi_{HS}\left\{n_{\alpha }\left(R,z\right)\right\}$ is independent of the number components *M*. It is the weighted densities $n_{\alpha }\left(r\right)$ exhibit such a dependence and are defined by

(10) $$\begin{array}{c} n_{\alpha }\left(r\right)=\sum_{i=1}^{M}\int d^{3}r^{\prime }\rho_{i}\left(r^{\prime }\right)\omega_{\alpha }^{i}\left(r-r^{\prime }\right)), \end{array}$$

and the weighting functions $\omega_{\alpha }^{i}\left(r\right)$ are

(11) $$\begin{array}{c} \omega_{3}^{i}\left(r\right)=\Theta \left(R_{i}-r\right),\;\omega_{2}^{i}\left(r\right)=\delta \left(R_{i}-r\right),\;\omega_{1}^{i}\left(r\right)=\frac{\omega_{2}^{i}\left(r\right)}{4\pi R_{i}},\;\omega_{0}^{i}\left(r\right)=\frac{\omega_{2}^{i}\left(r\right)}{4\pi R_{i}^{2}},\omega_{2}^{i}\left(r\right)=\frac{r}{r}\Theta \left(R_{i}-r\right),\\ \omega_{1}^{i}\left(r\right)=\frac{\omega_{2}^{i}\left(r\right)}{4\pi r_{i}}. \end{array}$$

Here Θ and δ denote the Heaviside step function and Dirac delta function, respectively. The long-range contribution to the free energy functional is

(12) $$F_{long}^{ex}\left[\left\{\rho_{i}\left(z\right)\right\}\right]=\frac{\pi }{2}\sum_{i=1}^{M}\sum_{j=1}^{M}\int RdR\int dz\times \int dz^{\prime }\rho_{i}\left(R,z\right)\rho_{j}\left(R,z^{\prime }\right)\;\Phi_{LR}(R,|z-z^{\prime }\left|\right),$$

where $\Phi_{LR}(R,|z-z^{\prime }\left|\right)$ is the long-range interactions contribution of the reduced free energy. Equation (12) indicates that the long range interactions are accounted for in a mean-field limit [23]. The term $F_{SC}\left[\left\{\rho_{i}\left(z\right)\right\}\right]$ refers to the free energy due to ion-ion correlations and is discussed in detail below.

The last term in Equation (6) is the Lagrangian constraint, which accounts for the external fields $V_{i}^{ext}$ and fixed chemical potentials $\mu_{i}$ for all species.

The densities of all components $\rho_{i}\left(z\right)$ are found by minimizing the functional [15,24]

(13) $$\begin{array}{c} \frac{\delta \Omega \left[\left\{\rho_{i}\left(z\right)\right\}\right]}{\delta \rho_{i}\left(z\right)}=0. \end{array}$$

which produces the so-called Euler-Lagrange equations. Typically, these are solved for the unknown component profiles, $\rho_{i}\left(z\right)$, by an iterative method.

The charge density distribution in the EDL is derived by summing over the individual charged species, taking into account the charge numbers with their sign

(14) $$\begin{array}{c} \rho_{e}\left(z\right)=\sum_{i=1}^{M}q_{i}\rho_{i}\left(z\right). \end{array}$$

This approach includes the contributions of the interactions between the solutions species, which are present in the terms $F_{HS}^{ex}\left[\left\{\rho_{i}\left(z\right)\right\}\right]$ [21,22], which accounts for the excluded volume effects and $F_{long}^{ex}\left[\left\{\rho_{i}\left(z\right)\right\}\right]$, which captures long-range interactions. In our model, these interactions are of the Lennard-Jones (LJ) [25]

(15) $$\begin{array}{c} \Phi_{\mathrm{LJ}}\left(r_{ij}\right)=4\epsilon_{ij}\left[{\left(\frac{d_{ij}}{r_{ij}}\right)}^{12}-{\left(\frac{d_{ij}}{r_{ij}}\right)}^{6}\right],r_{ij}>d_{ij} \end{array}$$

and Coulombic

(16) $$\begin{array}{c} \Phi_{\mathrm{el}}\left(r_{ij}\right)=\frac{q_{i}q_{j}}{4\pi \varepsilon \varepsilon_{0}r_{ij}},r_{ij}>d_{ij}, \end{array}$$

type [14,17,18,19,20], where $d_{ij}=\left(d_{i}+d_{j}\right)/2$, $d_{i}$ is the diameter of component “*i*” and $r_{ij}\left(R,z\right)$ is the distance between species “*i*” and “*j*”. All non-Coulombic interactions of a molecule (or ion) of type “*i*” with the interface are assumed to be of the “hard wall” type, that is, only the excluded volume effects are taken into account. The only exception is our analysis of the solvent-wall interactions presented in Section 3.3 below, where the LJ (9-3) potential [25]

(17) $$\begin{array}{c} \Phi_{\mathrm{LJ}}\left(z\right)=\epsilon_{s-w}\left[\frac{2}{15}{\left(\frac{d_{s}}{z}\right)}^{9}-{\left(\frac{d_{s}}{z}\right)}^{3}\right],z>d_{s}/2 \end{array}$$

is used in the analysis. The electrostatic interaction of the ions with the interface is

(18) $$\begin{array}{c} \Phi_{\mathrm{el}}\left(z\right)=\frac{q_{i}\sigma z}{2\varepsilon \varepsilon_{0}},z>d_{i}/2. \end{array}$$

Other choices for the attractive interactions are also available. For example, Oleksy and Hansen [26,27,28] used a Yukawa potential to account for the long-range attraction between the solution species.

## 4. Higher Order Electrostatic Correlations

In the results presented so far, the electrostatic contribution to the energetic components in the cDFT formalism has been treated only at the mean field level; that is, no electrostatic correlations between the ions have been included except that they feel the average electrostatic potential. However, higher order electrostatic correlations beyond the mean field can be quite significant. This is especially true for multivalent ions but these correlations also play a role for monovalent ions. In this section, we review the origin and effect of these higher order electrostatic correlations.

To give an intuitive description of the origin of the electrostatic correlations, consider a homogeneous (bulk) electrolyte solution. There, the ions interact via the Coulomb potential, perhaps moderated by the dielectric constant of the solvent. Because the system is homogeneous, the mean electrostatic potential is identically zero; averaging over a sufficiently long period of time, there is charge neutrality everywhere. However, the nanoscale structure of how the ions arrange around each other (i.e., how the screen each other) depends strongly on the electrolyte concentration and the charges on the ions. Specifically, the length scale of screening decreases as either the concentration or the ion valences increase. This is reflected in the Debye length, the most commonly used estimate of the screening length. If ions are generally closer to each other when they are at high concentration or when one species is multivalent and this is not reflected in the mean electrostatic potential, then there must be an energy term in the cDFT formalism (Equation (6)) that accounts for these correlations between the ions. We call this the screening term, denoted with SC.

Virtually all modern cDFT formulations of the screening energy term are based on the Mean Spherical Approximation (MSA). The MSA is a theory of homogeneous electrolytes that extends the classical Debye-Hückel theory to include the size of the ions [29,30,31]. Other theories like the Hypernetted Chain approach are more accurate [31] but the MSA has the advantage of explicit analytic formulas, which makes it particularly convenient to generalize it to inhomogeneous electrolytes in cDFT.

Here, we do not focus on different cDFT formulations that have been created to approximate this term but rather the effect the screening term has on EDL structure. A number of different expressions have been derived for the screening term over the last 30 years. These provide different levels of trade-off between accuracy (EDL structure compared to Monte Carlo simulations) and computational speed. For the results shown here, we use the Reference Fluid Density functional of Gillespie et al. [32,33], which remains the most accurate one to date. Other cDFT formulations include the bulk reference Taylor expansion [34,35] and the functionalized MSA [36]. The relative accuracies of these three approaches was recently catalogued [37].

## 5. Molecular Interactions, Solvent Polarity Effects and Dielectric Permittivity

Electric double layers typically form at interface of substrates with electrolyte solutions. Hence, the Coulombic interactions that involve the ionic species are of primary importance. However, the existence of a stable liquid phase requires the presence of attractive forces forces between the solvent molecules. The solvent molecules also interact with ions, which accounts for the ionic solvation. Depending on the solvent polarity, its molecules may be involved in isotropic attractive (e.g., Lennard-Jones, Yukawa, etc.) or dipole interactions. In addition, all species have finite size and that leads to a short-ranged repulsive force, which plays a major role for the liquid structure at the molecular scale.

A simple description of electrolytes and EDLs can be accomplished by ignoring all interactions except for the Coulombic (and in some cases the short-range repulsion) between the ionic species. Such models are colloquially referred to as “primitive” [38]. The primitive models [36,39,40,41,42,43,44] consider the solvent to be a structureless continuum, characterized by a uniform relative dielectric permittivity $\varepsilon_{r}$. The model reflects the ionic interaction contributions but fails to capture the structural effects caused by the presence of the neutral solvent. At the opposite extreme are the “civilized” models [45,46,47,48,49,50,51,52,53] that account for the presence of all solutions components and all possible interactions between them—Coulombic, van der Waals, dipole-dipole, ion-dipole and so forth. The inclusion of the dipole effects, that are due to the solvent polarity, requires an additional integration to properly average all dipole orientations. Such a step needs significantly greater computational resources and time. A reasonable compromise is offered by the “semi-primitive” models, which explicitly account for the presence of solvent molecules but ignore the effects due to the their polarity. This approximation means that the solvent molecules exhibit short-range repulsive forces and isotropic attractions (necessary to ensure the existence of a stable liquid phase) but are not involved in any orientation-dependent dipole-dipole or ion-dipole interactions. The semi-primitive models proved themselves helpful in demonstrating the effect of the liquid structure on the properties of charged electrolyte interfaces [14,17,18,19,20,54,55,56,57,58,59]. Since the semi-primitive approach does not account for any dipole effects, the dielectric permittivity has to be added *ad hoc* in order to properly scale all Coulombic terms. A very insightful analysis of the problem was recently offered by Oleksy and Hansen [26,27], who argued that this approximation is quite reasonable and can be further improved by introducing a dielectric permittivity that depends on the local weighted solvent density ${\tilde{\rho }}_{0}\left(z\right)$ defined by

(19) $${\tilde{\rho }}_{0}\left(z\right)=\frac{6}{\pi d_{0}^{3}}\int_{0}^{\infty }dz^{\prime }\rho_{0}\left(z^{\prime }\right)\left[\frac{d_{0}^{2}}{4}-{\left(z-z^{\prime }\right)}^{2}\right]\Theta (\frac{d_{0}}{2}-|z-z^{\prime }\left|\right).$$

The local weighted solvent density density can then be used to calculate the local dielectric permittivity $\varepsilon_{r}\left(z\right)$. An example is the Clausius-Mossotti equation

(20) $$\varepsilon_{r}\left(z\right)=\frac{\left(8\pi /9k_{B}T\right)m^{2}{\tilde{\rho }}_{0}\left(z\right)+1}{\left(4\pi /9k_{B}T\right)m^{2}{\tilde{\rho }}_{0}\left(z\right)-1},$$

where *m* is the molecular dipole moment. The Clausius-Mossotti equation can be derived using a mean-field cDFT model of dipolar hard sphere fluid [28]. Unfortunately it fails for high dipole moments and cannot be used for liquids such as water [27,28]. For such cases, Oleksy and Hansen [26,27] proposed the empirical expression

(21) $$\varepsilon_{r}\left(z\right)=1+\frac{f(T)}{1+\exp[-a\left({\tilde{\rho }}_{0}\left(z\right)d_{0}^{3}-\rho_{0m}\left(T\right)d_{0}^{3}\right]}.$$

The function $\rho_{0m}\left(T\right)=\left[\rho_{0}^{g}\left(T\right)+\rho_{0}^{l}\left(T\right)\right]/2$ is the mid-point fluid density between coexisting gas and liquid phases [with densities $\rho_{0}^{g}\left(T\right)$ and $\rho_{0}^{l}\left(T\right)$, respectively], while ${\tilde{\rho }}_{0}\left(z\right)$ is given by Equation (19). The empirical function f(T)=88−0.37T, was designed to conform to experimental data for water permittivity.

The Oleksy-Hansen approach is particularly useful when the semi-primitive cDFT model is applied to wetting electrolyte liquid films in coexistence with a gas phase. In such cases, the dielectric permittivities in the liquid and gas phases may vary by more than an order of magnitude and that is adequately captured by Equation (21) above. This model was tested against the civilized cDFT model proposed by Biben et al. [52], which fully accounted for the dipole effects at the molecular level and showed a very good agreement. Single phase liquids are less challenging since the dielectric permittivity does not experience such abrupt changes. Still, the local dielectric permittivity in a liquid solution is perturbed by the presence of charge (i.e., an ion or charged group.) This perturbation decays with distance and after a few molecular diameters the permittivity resumes its bulk value [51,52]. Hence, the detailed solvent polarity effect on the local dielectric constant may be neglected for low to moderately concentrated electrolyte solutions. For example each two ions in a 0.01 M solution are separated, on the average, by more than 30 solvent molecules, which is more than sufficient distance for the permittivity to relax to its bulk value. At higher electrolyte concentrations and/or in the presence of multivalent ions the local variations in the local dielectric permittivity should be taken into account either using the Olesky-Hansen approach (see Equation (21)) or by developing a full-scale civilized model including all possible interactions (van der Waals, Coulombic, dipolar, etc.). Another feature of concentrated electrolyte solutions is that the ionic screening correlations become important and need to be properly taken into consideration [36,44].

## 6. Results and Discussion

### 6.1. Solvent Effects

**Figure 1:**
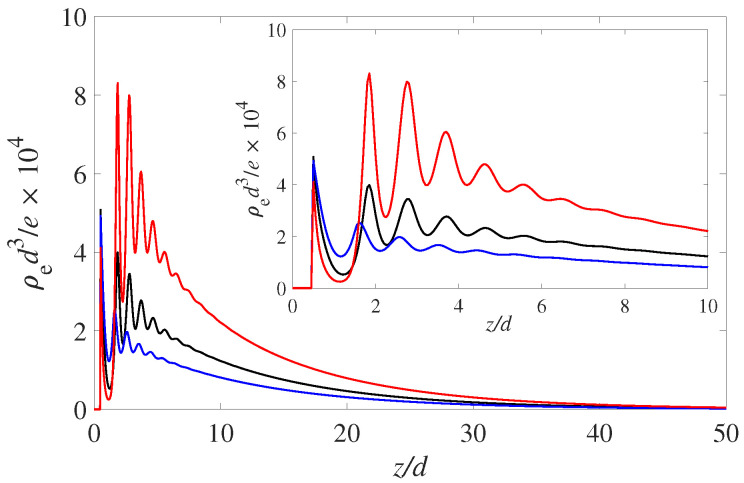
Fluid charge density $\rho_{e}$ for three different cases of solvent-wall interactions: the blue curve is for $\varepsilon_{s-w}=0$ (solvophobic), the black is for $\varepsilon_{s-w}=1k_{B}T$ (neutral, where the energy is the same as the bulk LJ attraction). The red curve is for $\varepsilon_{s-w}=2k_{B}T$ (solvophilic).

As an illustration of the importance of including the solvent in cDFT we consider an EDL in a situation where the solvent is attracted to the wall with three different wettability conditions. Thus, we mimick a solvophilic wall, a solvophobic wall and a partially wetting wall that we will refer to as a neutral wall. The wetting variations are accomplished by varying the strength of the LJ interactions (i.e., through $\varepsilon_{s-w}$—see Equation (17)) between the wall and the solvent component. We vary $\varepsilon_{s-w}/k_{B}T$ from 0 to 1 to 2. Note, that for the ion species the value is held constant at 1 throughout. Although we can be sure that the case $\varepsilon_{s-w}/k_{B}T=0$ produces complete drying at liquid-vapor coexistence, the other two values are merely inspired guesses and we are not implying that (at liquid-vapor coexistence) they would produce complete wetting for $\varepsilon_{s-w}/k_{B}T=2$ or a contact angle of 90 degrees for the neutral wall. The results for the spatial net charge distributions are shown in Figure 1. These are the results for a symmetric monovalent electrolyte at fairly low molarity (0.01 M). The bulk pH is set at 4 and the charge regulation parameters are set at $pK_{+}=-2$ and $pK_{-}=6$.

The solvophilic (red) curve, $\varepsilon_{s-w}/k_{B}T=2$, shows the most pronounced build-up of positive charge variation in the EDL. Note that all three wetting states display a pronounced layering oscillation with a period of 1 solvent diameter. As mentioned this is due to the packing of the solvent molecules. The increased number of solvent molecules at the wall prevents the PDIs form approaching and neutralizing the negative surface charge. Hence, the more negative interface attracts the positive counterions in the EDL. This layering modulation is carried over to the positive and negative ions and hence is reflected in the profiles of the net charge ($\rho_{e}\left(z\right)$).

**Figure 2:**
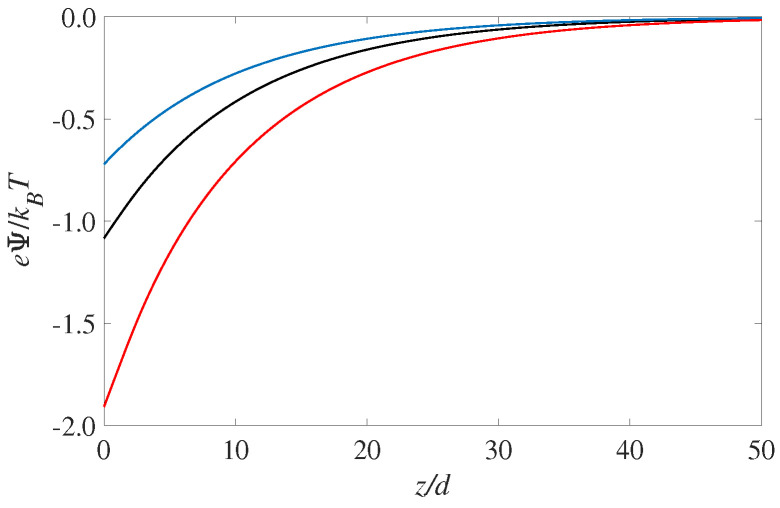
Electrostatic potential for three different cases of solvent-wall interactions: the blue curve is for $\varepsilon_{s-w}=0$ (solvophobic), the black is for $\varepsilon_{s-w}=1k_{B}T$ (neutral, where the energy is the same as the bulk LJ attraction). The red curve is for $\varepsilon_{s-w}=2k_{B}T$ (solvophilic).The results shown correspond to the same charges as for Figure 1.

The corresponding profiles for the electrostatic potential is shown in Figure 2. As expected the largest value for the surface potential (i.e., the potential value at z=0) occurs for the solvophilic case, $\varepsilon_{s-w}/k_{B}T=2$. It is interesting to see that the electrostatic potential is monotonic throughout the EDL. That is, there is there are no hints from the layering seen in Figure 1. This is consistent with Poisson’s equation (Equation (1)), which shows that it is the second derivative of the potential that corresponds to the (net) charge distribution. Since the overall sign of the fluid charge density does not change, the potential curvature (or $d^{2}\Psi /dz^{2}$) will not change as well. The magnitude of the curvature, however, will change according to the the fluid charge density curves shown in Figure 1 but this is hard to notice by visually observing the potential curves in Figure 2. However, differentiating the potential curves twice will recover the oscillating results for the fluid charge density $\rho_{e}\left(z\right)$ shown in Figure 1.

The results shown here are a demonstration of the role of including an explicit solvent in cDFT. It is clear that the solvent’s presence is necessary to capture the effects of different wettabilities of the solid phase. Moreover, to capture thin wetting films it is essential to include the formulation of a density dependent dielectric permittivity described in the previous section.

### 6.2. Effect of Screening Correlations on EDL Structure within the Primitive Model

The vast majority of the studies studying the effect of the screening term were performed with the primitive model of ions, where the ions are charged hard spheres and the solvent is a background dielectric material; there are no explicit water particles, as there are for the other results shown in this article. Therefore, it is not known exactly how the inclusion of higher order electrostatic correlations will affect these results but a number of generalities will carry over. The focus of this section will be discussing these general properties but within the framework of the primitive model of electrolytes. While many of these properties have been generally known for a while, how they all come together to define EDL structure was only recently described in detail by Voukadinova and Gillespie [44].

In the primitive model, ion concentration profiles as a function of the distance *x* from a smooth, hard, uniformly charged (as opposed to the charge regulated approach described above) surface are given by

(22) $$\rho_{i}\left(x\right)=\rho_{i}^{b}\exp\left(\frac{-\Delta \mu_{\mathrm{HS}}^{i}\left(x\right)-q_{i}\Delta \Psi \left(x\right)-\Delta \mu_{\mathrm{SC}}^{i}\left(x\right)}{k_{B}T}\right),$$

where *i* is the ion species. Here, we assume that the surface charge is negative and *constant* (i.e., not regulated). The three terms in the Boltzmann factor are different components of the interaction physics. The first term (HS) is the hard-sphere contribution, and $\Delta \mu_{\mathrm{HS}}\left(x\right)$ is the energy it takes to insert an uncharged ion at distance *x*, relative to the bulk value far from the wall. Similarly, $\Delta \Psi \left(x\right)$ is the mean electrostatic potential relative to bulk. The third term is the screening term. All these terms are the functional derivative of a corresponding cDFT free energy term, see Equations (6) and (13).

To see the effect of the screening term most clearly, we rewrite this to focus on the electrostatic potential profile. Specifically, we write this in terms of the counterion (cation) species but for convenience drop the species notation:

(23) $$q\Delta \Psi \left(x\right)=-k_{B}T\ln\left(\frac{\rho \left(x\right)}{\rho^{b}}\right)-\Delta \mu_{\mathrm{HS}}\left(x\right)-\Delta \mu_{\mathrm{SC}}\left(x\right).$$

In this equation, the first two terms on the right-hand side are negative. The first term is negative because, when the surface charge on the wall is large enough, the cation concentration will be greater than the bulk value far from the wall. This also causes the second (HS) term to be negative because the more dense an area is, the more difficult is to insert an uncharged particle. The SC term, on the other hand, is almost always negative [44] because, just like in a homogeneous system, having higher ion concentrations makes the screening energy more negative. This makes the third term positive.

In the classical Poisson-Boltzmann theory where the HS and SC terms are ignored, the electrostatic potential given just by the first term in Equation (23). Therefore, the balance between the HS and SC terms determines how the electrostatic potential is different from the classical case. In their recent work, Voukadinova and Gillespie [44] found that at low to moderate service charges the SC term in Equation (23) is more positive than the HS term is negative; the balance is only flipped at extremely high surface charges when the counterion concentration is so large that it becomes extremely difficult to find space for more ions. Therefore, the effect of the higher order electrostatic correlations is to make the electrostatic potential less negative than the classical Poisson-Boltzmann case.

In fact, the screening term can become so large as to make the electrostatic potential positive. This is known as charge inversion. Charge inversion occurs mostly for multivalent ions and this is because the screening term scales as the square of the counterion valence [36]. Thus, the screening term becomes significantly larger the higher the valence.

**Figure 3:**
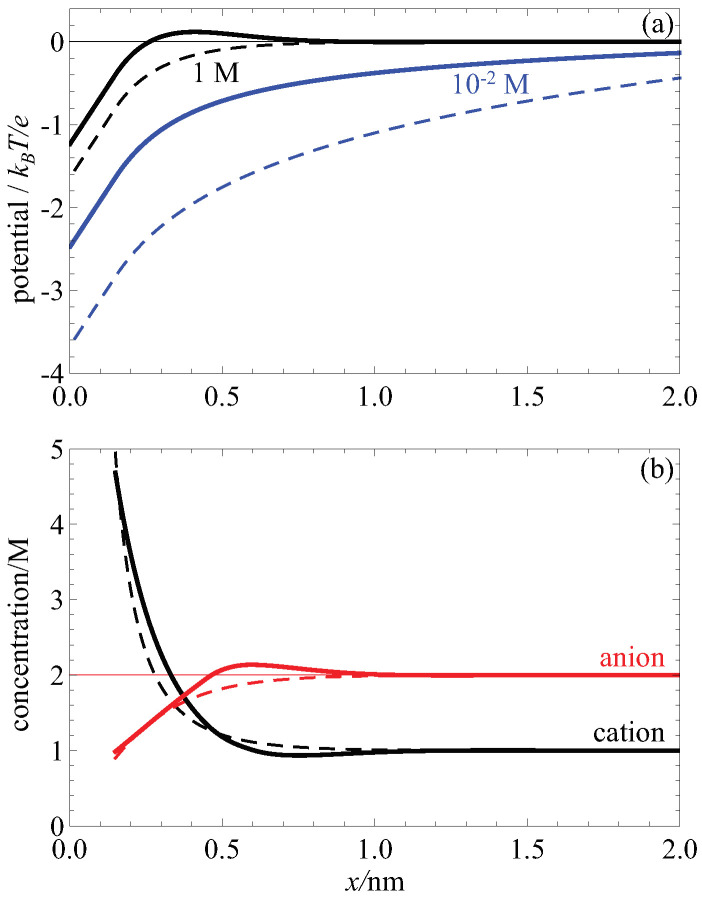
Classical density functional theory (cDFT) (**solid lines**) and Poisson-Boltzmann (**dashed lines**) calculations of electric double layer (EDL) structure for a primitive model electrolyte with +2 valence cations and −1 anions, both with 0.3 nm diameter. The surface charge is −0.1 C/m${}^{2}$. (**a**) Electrostatic potential versus *x*, the distance from the wall, for low ($10^{-2}$ M, blue lines) and high (1 M, black lines) bulk concentration. (**b**) Cation (**black lines**) and anion (**red lines**) concentration profiles for the 1 M case.

The effect of the screening term in making the electrostatic potential less negative than the Poisson-Boltzmann case and leading to charge inversion is shown in Figure 3a. There, the onset of charge inversion is shown by increasing the bulk concentration of an electrolyte with divalent cations. At low concentrations (blue lines), the electrostatic potential with higher order electrostatic correlations (solid lines) is less negative compared to Poisson-Boltzmann without these correlations (dashed lines). This trend continues at high concentrations (black lines) but the potential with screening correlations becomes positive (solid line). Because the Poisson-Boltzmann theory does not include this negative contribution it can never have a change of sign in the electric potential.

This positive electrostatic potential has two consequences. Right now we focus on the consequence for the EDL structure; below we consider the effect on fluid flow. Specifically, the positive potential draws in anions (co-ions) in a second layer of ions behind the initial high concentration layer of cations (counterions). This is shown in Figure 3b. There, the anion concentration (solid red line) increases above the bulk concentration (thin red line); for the Poisson-Boltzmann case (dashed red line) this does not happen.

This negative-positive-negative sandwich of surface charge, counterions and co-ions is a hallmark of charge inversion and it is due to the change of sign of the electrostatic potential caused by the higher order electrostatic screening correlations. This is the most extreme consequence of these correlations but in general they make the electrostatic potential less negative and therefore tend to decrease the cation concentration and increase the anion concentration, relative to the classical uncorrelated Poisson-Boltzmann theory.

### 6.3. Effect on Fluid Flow

**Figure 4:**
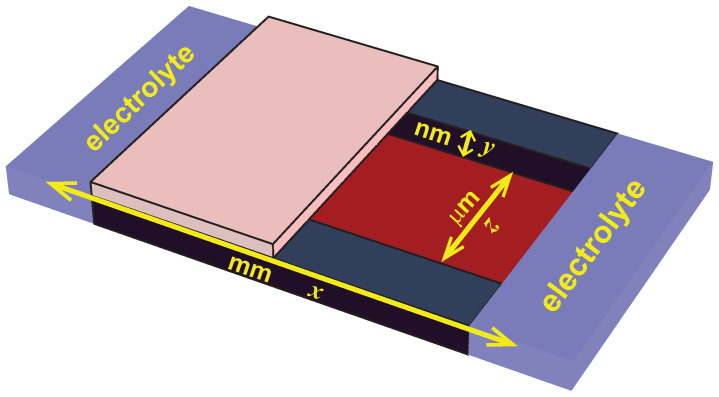
Geometry of a nanofluidic slit channel. Two wells with electrolytes (**blue**) are connected by a channel fabricated out of fused silica that is several millimeters in the *x* direction, several micrometers in the *z* direction and ∼100 nm in the *y* direction. The surface charge is on the top (**pink**) and bottom (**red**) of the device.

Beyond this effect of the electrostatic screening correlations on EDL structure, they and the concomitant charge inversion can also have a significant effect on fluid flow. This was explored in detail in References [60,61] for nanofluidic channels where electrolytes flow parallel to two charge surfaces that are of the order of 100 nm apart [62]. The geometry is illustrated in Figure 4.

In such a system, advection moves water through the channel based on the local mean electrostatic potential. (This movement of fluid is in addition to the movement of ions.) Specifically, the advective velocity profile at each longitudinal location *y* across the channel that drives fluid down the axial direction *x* of the channel is proportional to $\Psi \left(y\right)-\Psi \left(H_{S}\right)$ where $H_{S}$ is the slip plane location where the velocity is zero [63]. If one considers the velocity at the center of the channel where, for convenience, we take the electrostatic potential to be zero, then the direction of the velocity is given by the sign of $-\Psi \left(H_{S}\right)$. Consequently, if there is charge inversion and the slip plane is in the region a positive potential, then the fluid will flow in the opposite direction of when there is no charge inversion and also in the opposite direction of the ions.

Each of these has a consequence that can be measured in experiments. In the classical case without charge inversion, the counterions in the EDL and the water move in the same direction when a hydrostatic pressure gradient or electrostatic potential gradient is applied down the axial *x* direction of the channel. However, when charge inversion moves the fluid in the opposite direction of the ions, this reduces the total ion current (as some ions are in each volume of fluid that is moving) and can even change the sign of the current. These were the first measurable consequences of charge inversion in nanofluidics [64,65] and can be reproduced by cDFT [66,67].

In more recent work [60], the two wells at the end of the channel contained different electrolytes, one that exhibited charge inversion and one that did not. When an electrostatic potential was applied between the two wells that moves the two fluids towards each other (as the charge inverting fluid moves in the opposite direction of the other fluid), a stable front between the two fluids is established. Moreover, at this junction, low-concentration analyte ions will accumulate so that they can be pre-concentrated for analysis later. While the explanation of the physics of both the stable front and the ion accumulation is beyond the scope of this paper (see References [60,61]), this practical application is a macroscopic manifestation of the charge inversion produced by the higher order electrostatic screening correlations.

## 7. Conclusions

EDLs are inherently complex interfacial structures involving a multicomponent fluid with chemical ionization reactions present at the surface. To gain insight into EDLs it is often helpful to also consider basic models, as we have set out to do in this paper. One important aspect concerns the electrostatic screening effects acting between charges. These have been successfully studied within the primitive model, where the solvent is structureless background fluid. However, the conclusions reached by using the primitive model are transferable. That is, the main effect that the electrostatic screening correlations make the electrostatic potential less negative will still apply when explicit water particles are included. Moreover, they will remain an important contributor to charge inversion. However, what the relative balance of the screening term and the other terms will be when water is included remains to be determined. Nanofluidic flow was presented an application of the relevance of charge inversion.

EDLs with charge regulation boundary conditions and an electrolyte that contains an explicit solvent component display different rich interfacial behavior. That behavior includes a detailed structure dominated by the neutral solvent molecules, that strongly influences where the charged ions will be positioned. The solution structure near the charged interface affects the surface chemistry and hence there is a strong coupling between the solvent-induced effects and the charge regulation (for more details see References [14,18]). As an example of solvent effects we have presented the results for three types of wetting situations: solvophilic, neutral and solvophobic. These wetting types were created by enacting different interaction strengths between the solvent and the wall (while keeping all other interaction fixed). We find that although only the interactions between the wall and the neutral solvent were varied, the charge and electric potential were directly affected.

In this paper we have exclusively focused on simple planar surfaces. However, we stress that the methods discussed here are not limited to these simple cases. In fact, they can and have been applied to biological problems such as as ion channels. An example is the work of Gillespie et al. on the ryanodine receptor ion channel, see for instance, Reference [68].

## Abbreviations

The following abbreviations are used in this manuscript:

EDL	Electrostatic double layer
cDFT	Classical Density functional theory
HS	Hard sphere
LJ	Lennard-Jones
MSA	Mean spherical approximation
HNC	Hypernetted chain
SC	Screening correlations
